# Scoring systems for Oral Lichen Planus used by differently experienced raters

**DOI:** 10.4317/medoral.21833

**Published:** 2017-08-16

**Authors:** Margherita Gobbo, Katia Rupel, Valentina Zoi, Giuseppe Perinetti, Giulia Ottaviani, Roberto Di Lenarda, Lorenzo Bevilacqua, Sook-Bin Woo, Matteo Biasotto

**Affiliations:** 1 Division of oral medicine and pathology, Dental Science Department, Trieste, Italy; 2 Division of Oral Medicine and Dentistry, Brigham and Women’s Hospital, Boston, Massachusetts, USA

## Abstract

**Background:**

Scoring systems have been widely used to evaluate the severity and activity of oral lichen planus (OLP). The aim of the present study was to compare two existing (one modified) scoring systems in the evaluation of OLP severity and correlation with pain. Three differently experienced raters were involved.

**Material and Methods:**

Consecutive patients with OLP were assessed for pain using the Visual Analogue Scale and examined at 10 intraoral sites before starting (T0) and three weeks after (T1) steroid therapy (Clobetasol). Three differently experienced raters evaluated photographs using two scoring systems designated White-Erosive-Atrophic (WEA) modified from an older WEA system (WEA-MOD) and Reticular-erythematous-Ulcerative (REU) systems. WEA-MOD Kendall’s W and interclass correlation coefficient were calculated and correlation between REU/WEA-MOD and pain was calculated using Spearman coefficient.

**Results:**

Most patients showed lesions on buccal mucosa (85-93,5%) and maxillary/mandibular gingivae (31,8-31,2%), predominantly reticular. At T0, Kendall-W coefficients of 0.89 and 0.74 were obtained for the REU and WEA respectively. At T1, Kendall-W coefficients of 0.83 and 0.58 were obtained for the REU and WEA respectively. Interclass correlation coefficient ranged from 0.87 to 0.90 for REU and from 0.58 to 0.87 for WEA. REU and WEA scores significantly decreased after therapy (*p*<0.000) as well as VAS (*p*<0.05). REU score showed correlation with VAS.

**Conclusions:**

All the raters achieved comparable measures using REU whereas WEA and WEA-MOD seem less reproducible. REU seems to correlate to disease activity and pain.

** Key words:**Oral lichen planus, scoring system, VAS, REU, WEA, rater.

## Introduction

Lichen planus (LP) is a chronic, non-infectious inflammatory, mucocutaneous disease that is associated with T-cell-mediated immunological dysfunction leading to basal cell destruction (1,2). It affects women 2 to 3 times more commonly than men on average between the 6th and 7th decade of life (3). Medication-induced lichenoid eruption, lichenoid contact reaction (4), erythematosus lupus (5), chronic graft-versus-host disease (6) and C hepatitis-associated LP (7) may be clinically indistinguishable from idiopathic oral lichen planus (OLP). OLP prevalence in Europe rates between 0.5% to 3.4% of the population (8).

The most common form of OLP is characterized by white hyperkeratotic papules and striae involving the buccal mucosa, tongue and gingiva bilaterally and symmetrically, often with erythematous (erosive) or ulcerative areas (9,10). Treatment is generally with topical or systemic corticosteroids or immunomodulating agents (3,11). In Italy, the estimated prevalence of malignant transformation is approximately 1,3% in men and 2,9% in women (12,13).

Several scoring systems have been developed to grade the severity of OLP (14,15). In the present study, two of them were employed. One that is frequently used was developed by Thongprasom *et al*, (16) based on the presence and extent of white striae, erythema and atrophy (designated WEA); the other was developed by Pibooniyyom *et al.* (17) and is based on reticulations, erythema and ulceration (designated REU). Other scoring systems have been proposed (18,19). Having a good scoring system that correlates disease severity with subjective symptoms allows for comparison between baseline evaluation of lesions and efficacy of treatment or progression of disease.

- The main objectives of the present study were:

• To evaluate for agreement between a modified WEA (WEA-MOD) and REU scoring systems, correlation between them, and agreement between differently-experienced raters;

• To evaluate if the modified WEA-MOD and REU scoring systems correlate with a visual analogue score (VAS) for pain.

## Material and Methods

- Clinical examination

Consecutive patients were recruited at the Division of Oral Medicine and Pathology (Ospedale Maggiore, Trieste) between September 2014 and 2016. Patients were suspected as having OLP if they had bilateral white reticulations of either the buccal mucosa and/or tongue, regardless of whether erythema and ulcers were present, patients with bilaterally symmetric erythema of the gingiva with/without striations. Patients with extra-oral involvement were sent to seek expert help for the management of cutaneous and/or genital lesions. Histopathological confirmation of OLP diagnosis was the fundamental entry criteria, regardless the presence of pain. A total of 50 patients (33 females and 17 males, mean age 64±14 years) were enrolled.

During the first visit (T0), demographic and clinical information were collected. Each patient was asked to score pain using a 0-to-10 Visual Analogue Scale (VAS) with “0” representing the absence of pain and “10” the worst pain ever. Each patient was photographed at 10 different intraoral sites: right/left buccal mucosa, upper/lower lips (considered as a whole), hard and soft palate (including tonsils), maxillary and mandibular gingiva, ventrum/dorsum of tongue, floor of the mouth. A 5-mm punch biopsy was performed and blood test was performed for gamma glutamiltranspepsidase (γ-GT) and transaminases (ALT, AST), hepatitis B and C antibodies, complete blood count.

All symptomatic patients (VAS>4) were instructed to use Clobetasol (Clobetasol Propionate Ointment USP, 0.05%) for four times a day for seven days and then at a reduced frequency that kept them comfortable. Clobetasol was spread over sore areas and patients were instructed not to drink or eat anything for an hour. A follow-up visit was performed after 3-to-4 weeks (T1) when a second VAS was registered.

The study was approved by ethical committee. All procedures performed in studies involving human participants were in accordance with the ethical standards of the institutional and/or national research committee and with the 1964 Helsinki declaration and its later amendments or comparable ethical standards.

- Scoring System Performing

Differently experienced raters in the field of Oral medicine who were not used to employ scoring systems for OLP performed evaluations. The main (expert) rater was a Professor in Oral Medicine; the intermediate rater was a second-year trainee in Oral Medicine; the inexpert rater was a fifth-year dental student. Raters were asked to perform REU and WEA scores for each photo/patient at T0 and T1 and were blinded as regard as the time-point they were evaluating.

The WEA scoring system is not site-based but an overall measurement. To make it comparable to the REU system, which is site-based, the authors have tested a modified version of the WEA system (WEA-MOD). Specifically, a 0-to-5 score following the WEA system was applied to each of the ten sites considered by REU. The final score was obtained by summing all the values. As such, the WEA-MOD would range from 0 to 50.

- Statistical Analysis

Statistical analysis was performed using SPSS (16.0 windows). For each scoring system, inter-observer agreement was assessed by the Kendall’s W coefficient. Moreover, to determine the degree of agreement between observers within each scoring system, an intra-class correlation coefficient (ICC) was used, and presented as mean and 95% confidence interval (CI). Both the Kendall’s W and ICC vary between zero (no agreement) and 1.0 (maximum agreement). In particular, values of 0.4 to 0.6 generally indicate moderate agreement, while values above 0.80 indicate almost perfect agreement. In a further analysis, the degree of correlation between the two scoring systems and within each observer was evaluated by the Spearman rank correlation coefficient, along with the calculation of the linear regression equation. A Spearman coefficient was performed to assess if REU/WEA-MOD correlated with VAS at T0 and T1, respectively. Wilcoxon test was used to assess improvement of VAS, as well as modification of REU and WEA-MOD before and after therapy.

## Results

Fifty patients were included. A predominance of female gender was reported (33 patients) and the mean age was 64±14years. Descriptive analysis is reported in  Table 1.

**Table 1:**
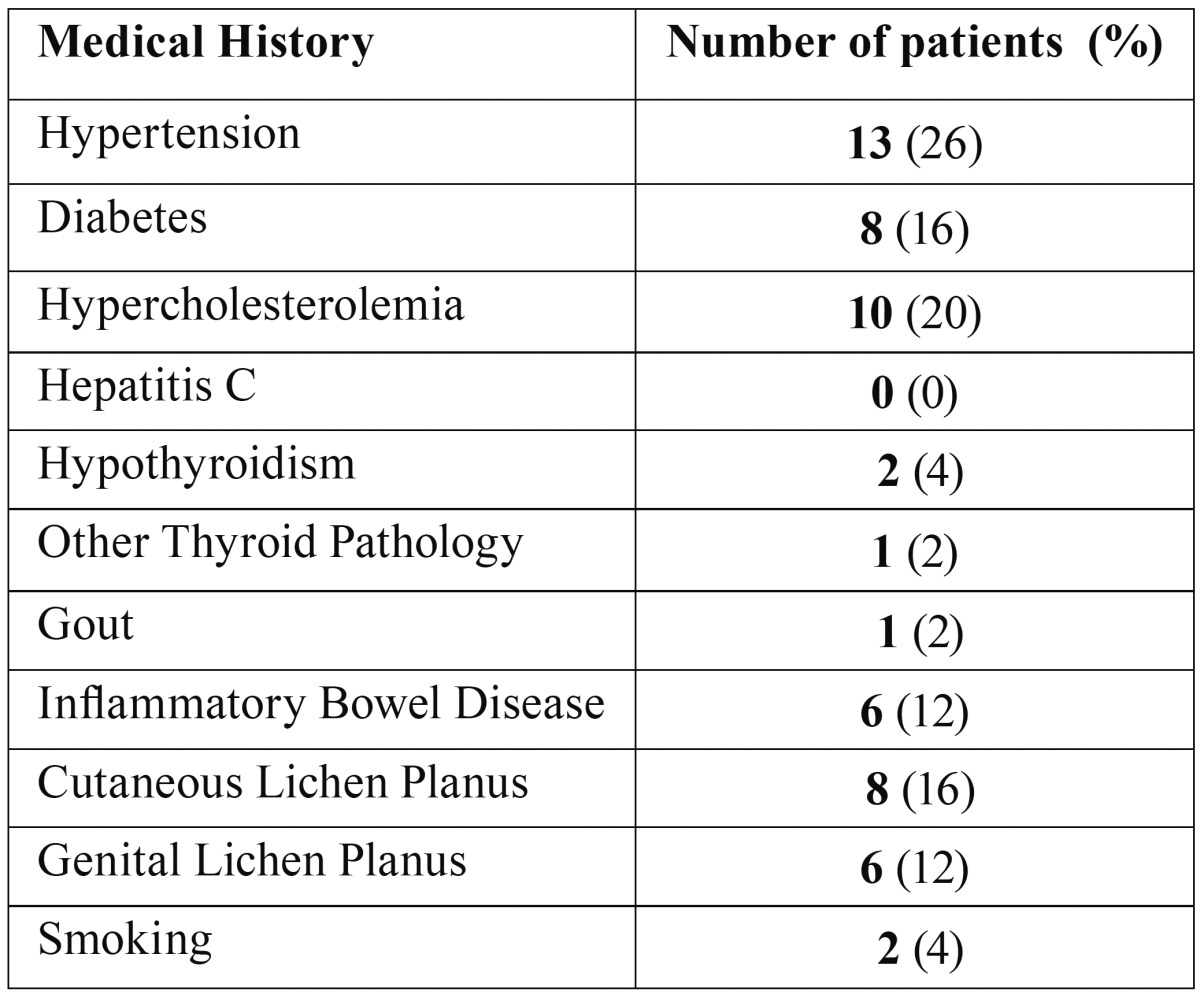
Descriptive analysis (50 patients) regarding distribution of analyzed pathologies.

The buccal mucosa was the most affected site, followed by maxillary and mandibular gingivae and tongue by both REU and WEA-MOD. According to REU, reticular lesions were the most frequent feature in all sites examined, followed by erythematous ones (Table  Table 2a). Ulcerative OLP was much less frequent, with the highest percentage found on bilateral buccal mucosa.

**Table 2A:**
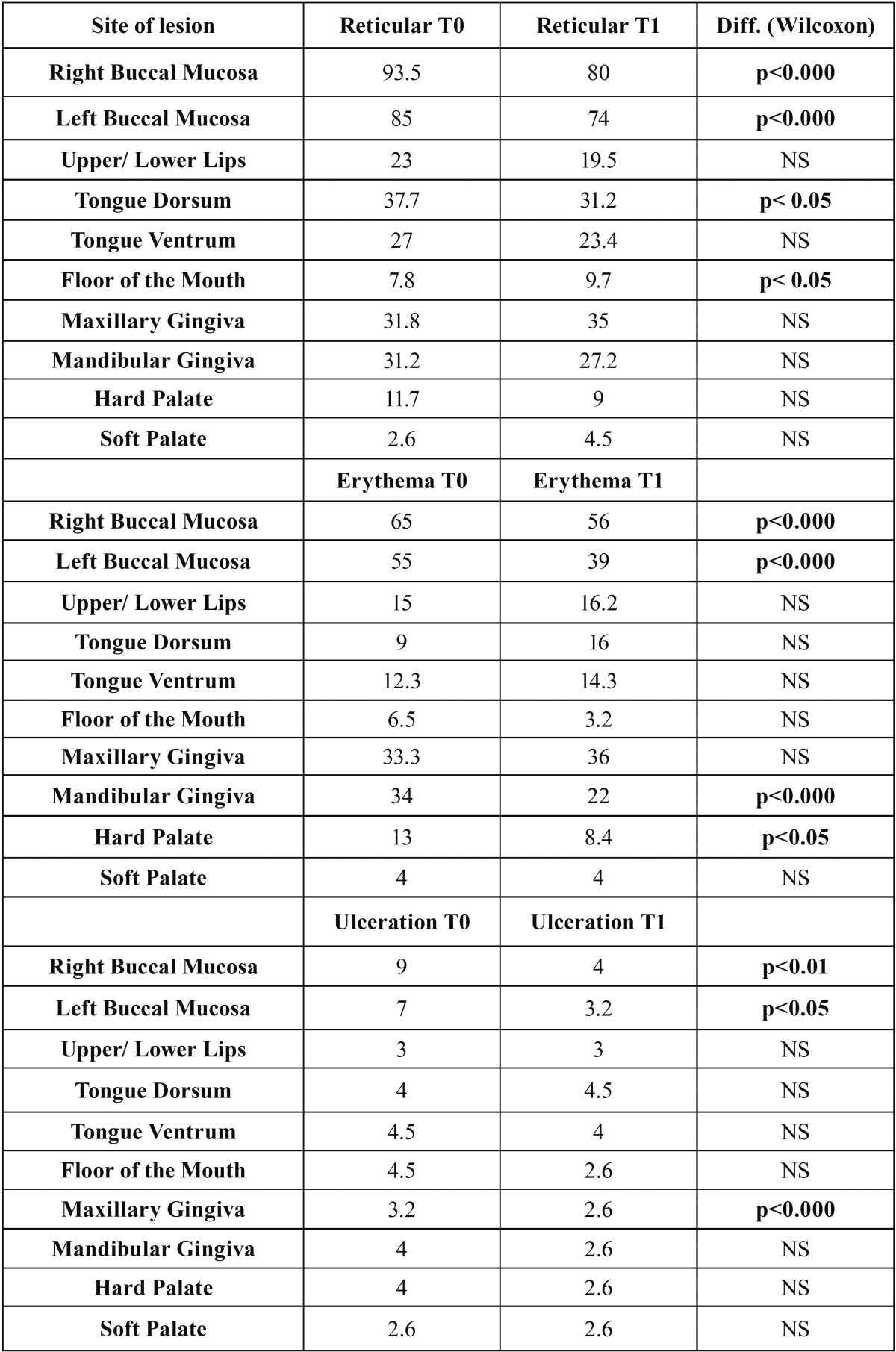
Distribution of lesions at T0 and T1 for REU. Values are expressed as percentages. For each OLP feature (Reticular, Erythema, Ulceration), absence vs presence has been considered. A *p* value less than 0.05 was used in the rejection of the null hypothesis.

Cutaneous and genital involvements were reported in 16% and 12% of patients respectively. In females, 15.2% reported genital involvement, 21.2% cutaneous involvement and 6.2% both. In males 5.9% had both, whereas none of the men had an isolated genital or cutaneous involvement.

Between T0 and T1, a decreasing of reticulated, erythematous and ulcerated areas was registered according to REU. A general improvement was also seen using the WEA-MOD scoring system (Table  Table 2b).

**Table 2B:**
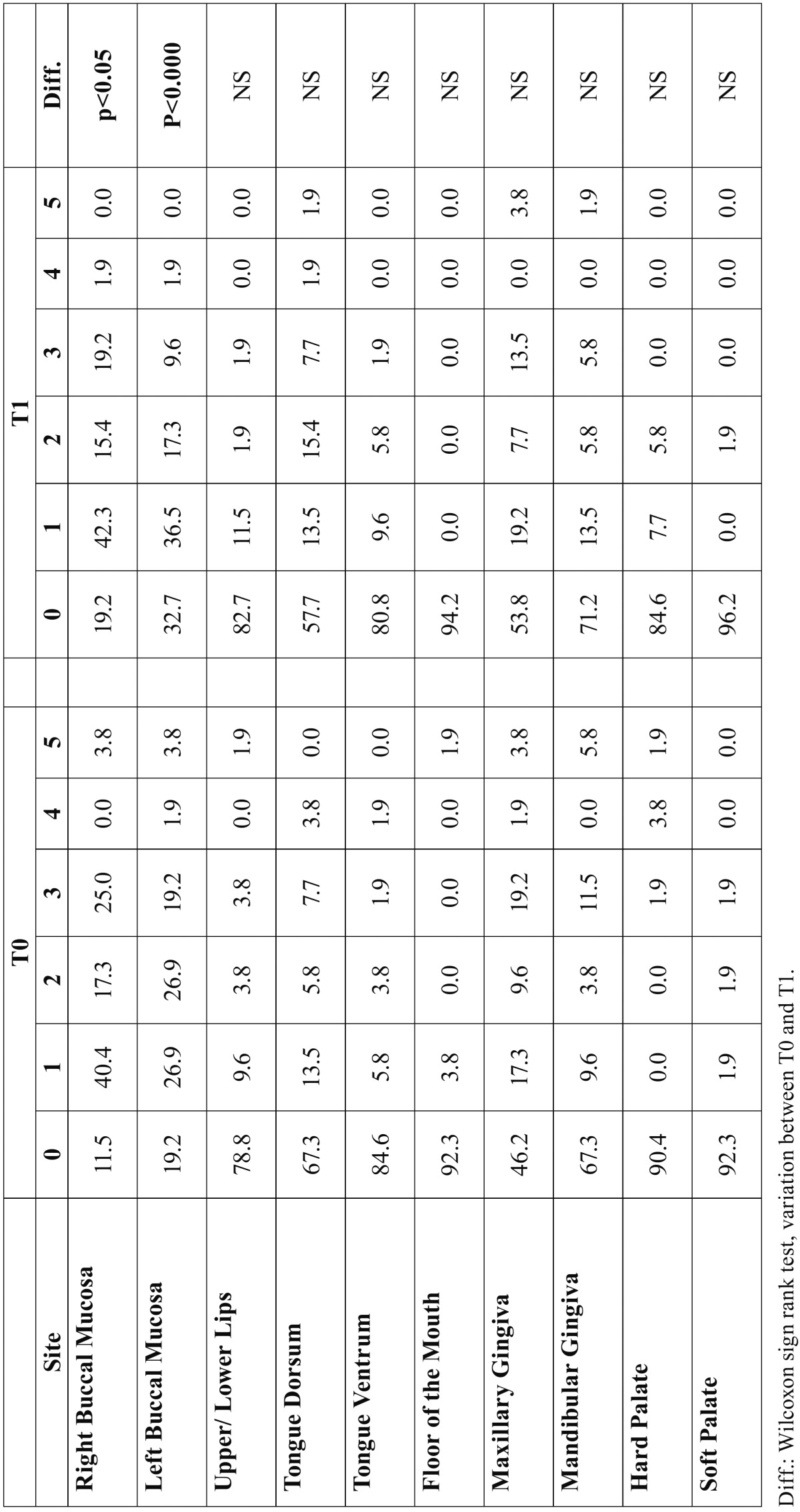
Distribution of lesions at T0 and T1 for WEA-MOD. For each score (0 to 5) values are expressed as percentages among valid cases. A *p* value less than 0.05 was used in the rejection of the null hypothesis.

Table  Table 2C shows the modification of OLP according to its severity (mild, moderate, severe) and to each scoring system.

**Table 2C:**
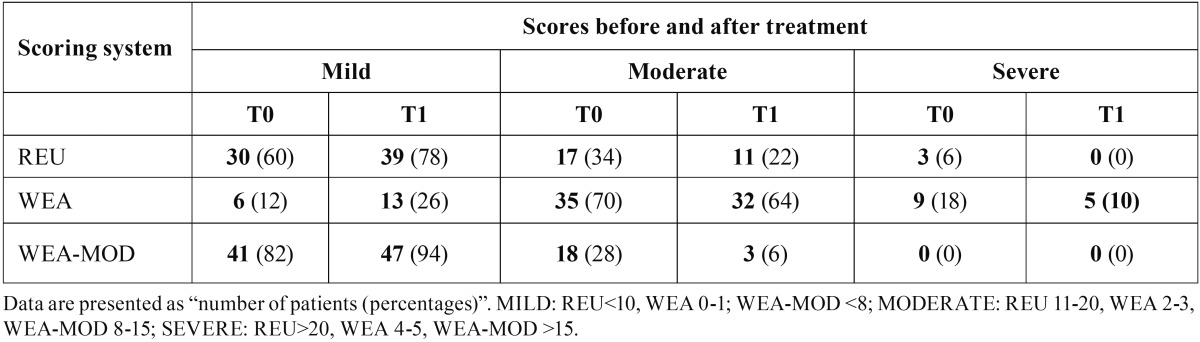
Scores before (T0) and after (T1) treatment for the REU, WEA and WEA-MOD (n=50).

Authors have evaluated variation of scoring system before and after therapy. Using the original WEA scoring system, changing of median value was not significant. In fact, a median WEA of 3 (IQR 1-5) was registered at T0 and of 2 (IQR 0-5) at T1. Using REU and WEA-MOD, a significant decreasing of the score was noted between T0 and T1 (*p*<0.000). Specifically, REU decreased from 8.3 (IQR 4.5-12.7) to 6.7 (IQR 3.6-9.7), whereas WEA-MOD decreased from 6.8 (IQR 3.9-9.7) to 5.7 (IQR 3.3-7.1).

**Figure 1:**
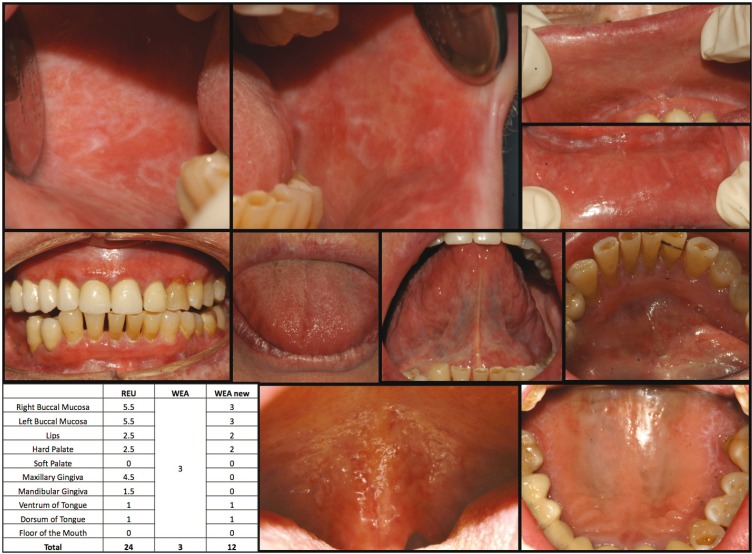
Show how the patients were scored using the REU and WEA-MOD.

**Figure 2:**
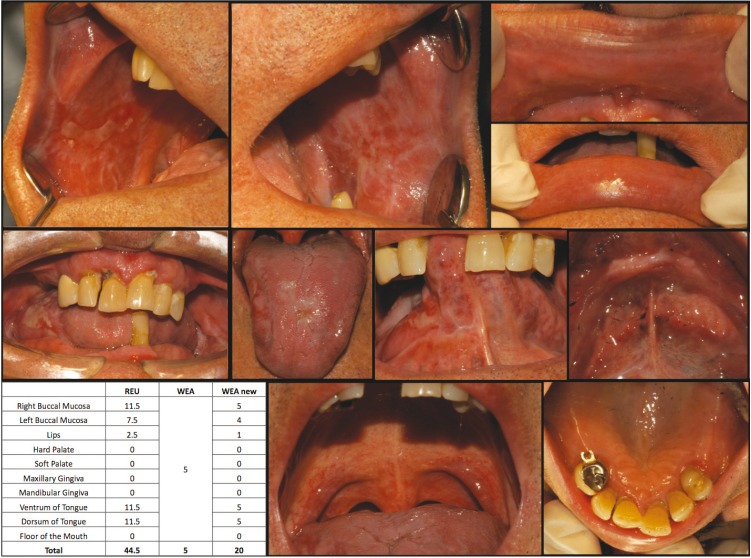
Show how the patients were scored using the REU and WEA-MOD.

Figure 1 and 2 show how the patients were scored using the REU and WEA-MOD.

 Table 3a reports the Kendall’s W coefficients for the REU and WEA-MOD scoring systems. Table  Table 3b reports the ICC, which was not applicable for the WEA since the range of REU and WEA-MOD scoring systems were too different.

**Table 3A:**
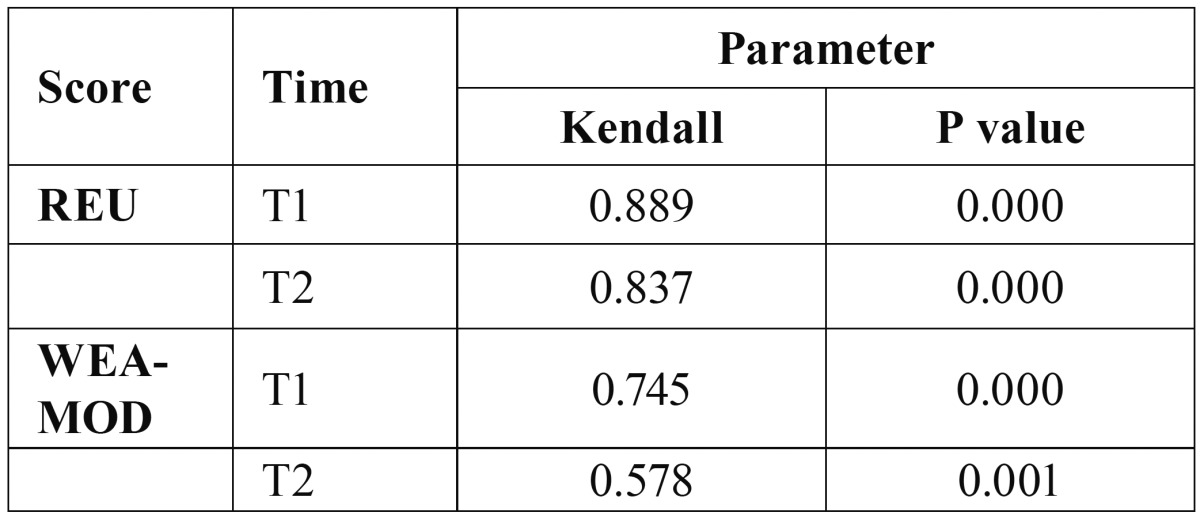
Kendall’s coefficient of concordance among the three operators (n=50), regarding REU and WEA-MOD.

**Table 3B:**
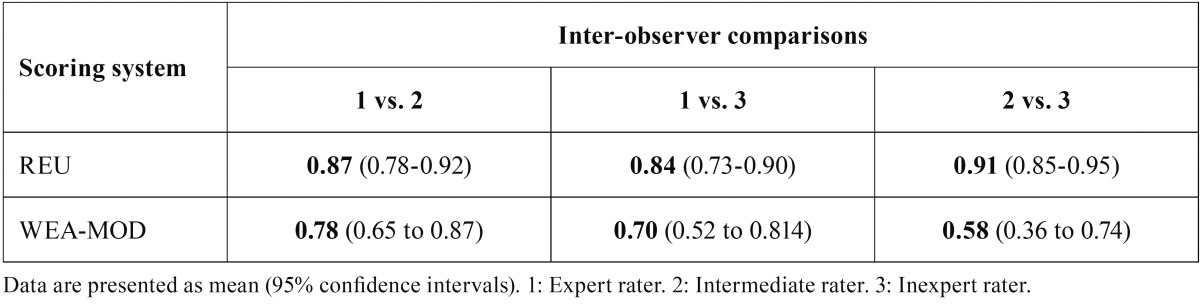
The intraclass correlation coefficients for the REU and WEA-MOD scoring systems between observers (n=50).

**Figure 3:**
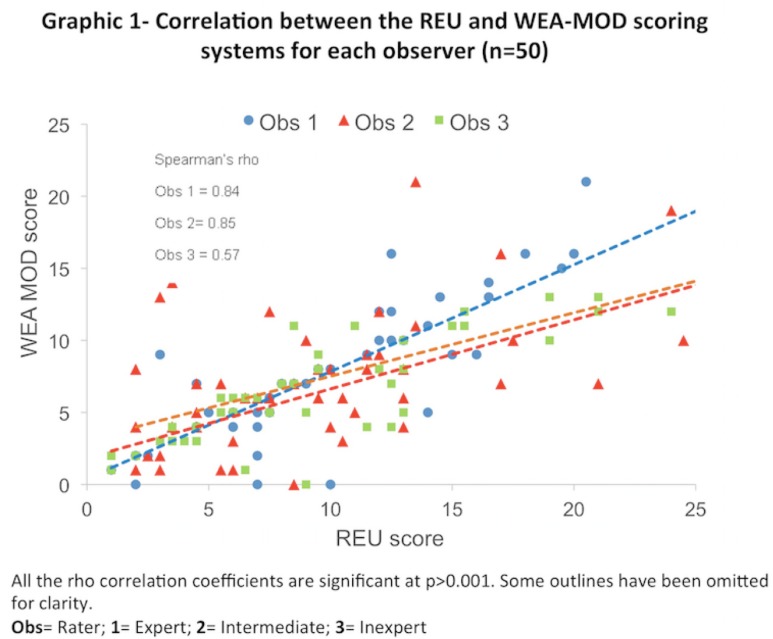
The degrees of correlation between the two scoring systems for each observer were 0.84, 0.85 and 0.57 for observers 1, 2 and 3, respectively.

The degrees of correlation between the two scoring systems for each observer were 0.84, 0.85 and 0.57 for observers 1, 2 and 3, respectively (Figure 3). However, observer 1 showed a greater increase in the WEA-MOD/REU score ratio for the highest values with slopes of the linear regression curve of 0.74, while the Observers 2 and 3 had slope values no less than 0.50.

Correlation with REU/WEA-MOD with pain is reported in Table  Table 3c.

**Table 3C:**
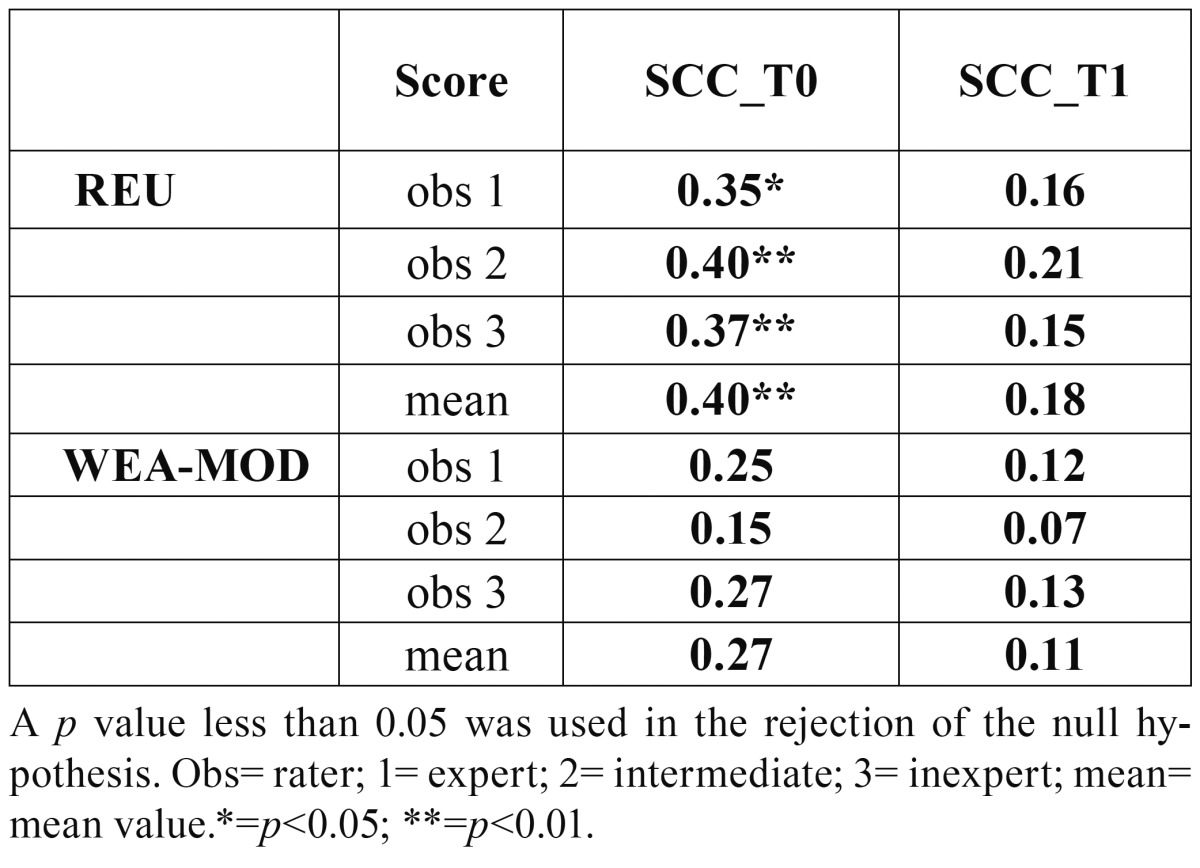
Correlation between VAS and REU/WEA-MOD through Spearman Correlation Coefficient (SCC). Values are calculated at T0 and T1.

## Discussion

LP is a mucocutaneous disease, often limited to the oral mucosa, although at various stages of severity and with several features. Although a specific antigen has not been identified yet, it is thought that OLP is a T-cell mediated process, where immune system targets the basal cells, in response to some antigenic changes in the oral mucosa, thus triggering apoptosis of the epithelial cells in the mucosa (20).

- Descriptive analysis and clinical findings

In our cohort of 50 patients, there was a 2:1 female: male ratio with mean age in the 7th decade, which is typical for OLP. Bilateral buccal mucosa, gingiva and tongue were the most commonly affected sites and this is also typical for OLP.

OLP has been correlated with HCV infections with a prevalence of 9.6% among 633 OLP sero-positive patients (21) to a high prevalence of 19.1 (22). In the present study, no patients were found positive for HCV. The reduced number of enrolled subjects does not allow us to state if this is related to differences in the prevalence of HCV in the North-eastern part of Italy or possibly ethnic differences. As regard as patients’ medical history, we have collected data on underlying diseases and we have compared their prevalence with that of general population ( Table 1). We have not found significant prevalence of analysed diseases in the OLP population (23).

- Scoring system evaluation

Several scoring systems have been developed for the evaluation of the severity of oral LP; newer ones are based on multiple sites and include pain scores. One of the oldest ones developed by Thongprasom *et al.* and referred to here as WEA, uses a simple 0-5 system. The REU system developed by Pibooniyom is more complex because it scores 10 sites and as such has a wider “spread” of scores (17). The system developed by Escudier *et al.* around the same time also uses multiple sites but is more complex to use because there are 17 sites and combines site scores with activity scores for a severity scores, but ratings are not uniform for each site (24). The one developed by Malhotra *et al.* uses multiple sites as well, but does not discriminate between keratotic, erosive or ulcerative disease (25).

In the present study, we compared WEA-MOD and REU scoring systems before and after treatment, and evaluated their correlation with pain. The unmodified WEA system evaluates the whole mouth as a single site and has limited utility. For example, the presence of erosion >1cm2 automatically gave the patient a score of 5, regardless of whether the erosion was at one site or 10. This does not always match with the severity of the disease all around the oral cavity and does not reflect the real extent of the pathology. It was also difficult to compare results using scores from WEA and REU because of the huge different in ranges between the two scoring systems. As such, we modified the WEA by using it for each of 10 sites to increase its comparability with REU (which is based on 10 sites) and its reliability. Also, since REU seemed more difficult to employ, we had hypothesized that WEA-MOD could increase reliability and decrease complexity of WEA and REU, respectively.

Concordance between WEA-MOD and REU was higher, yielding a Kendall’s W coefficient over 0.81. This means that both scores have a high reproducibility, although values were slightly higher for REU. While the REU score proved a very good ICC for all three raters, values were proportionally lower according to the experience of the rater. In fact, the expert and intermediate raters provided similar evaluations; the inexpert coefficient was fair to moderate. This value mirrors the learning curve of the techniques. The linear regression curve, suggests that WEA-MOD has a sharper learning curve than REU, meaning that REU may be easier to use (Fig. 3).

However, the criteria employed by REU are better defined and easier to apply than those employed by WEA and even the WEA-MOD. The REU system asks the observer to look for R (reticulated or papular, white) lesions, E (erythematous/erosive, red) lesions and U (ulcerated, yellow) lesions. The term “erosion” used in WEA may be interpreted as thinned and/or red and erythematous; there is no ulcerative category in WEA. The term “atrophy” refers usually to reduction in the thickness of epithelium or loss of differentiated structures. Epithelial atrophy also causes redness and this feature may be confused with erosion. For this reason, the inexperienced rater reported that he had difficulty in deciding on whether to score a lesion as erosive or atrophic. Consequently, although apparently easier, the WEA-MOD seems more confusing. Moreover, if we consider the learning curve, the difference between raters 2 and 3 is not huge so the score may be easily learnt and applied successfully after a little training. This is an important point of strength of the technique. Other advantages of REU over WEA may be highlighted. Firstly, it takes size into consideration and larger lesions have a higher score except for reticular and keratotic areas, which are scored absent (0) or present (1) only because they are not usually very symptomatic. Secondly, REU employs a weighted score based on ulcers being more symptomatic than erythema, which is more symptomatic than just keratotic lesions. This also allows a larger spread of the score. Thirdly, scoring all sites also allows for a larger spread of the score and a picture of the patients oral condition at a glance. For example, a score of R5E3U2 (weighted 12.5) clearly informs the clinician that there are reticulations at 5 sites, with some erythema and some ulcers.

Correlation between pain and REU score has been reported in the past and was also noted here (26). In fact, for all the raters a Spearman coefficient over 0.3 was obtained. This can let us conclude that the higher the value of REU, the worst pain reported. On the contrary, neither for WEA nor for WEA-MOD a correlation was evidenced. Consequently, the score could be used to mirror the symptoms of patients and to evaluate the efficacy of therapy. We can hypothesize that the other scores are more objective and cannot be considered a real reproduction of the feelings of the patient.

The correlation between a scoring system and referred pain is fundamental if you consider OLP as a chronic affection (27). In fact, patients affected by OLP usually experience pain only in some phases of the disease, followed by periods of remission. Therapy of OLP is based on topical or systemic steroids depending on the severity of the manifestations and pain (28). Knowing the exact dose of steroids the patient needs when experiencing certain levels of pain would be advantageous and would help the patient to self-regulate therapy (29).

The OLP is considered a precancerous condition with transformation ratio varying between 0 and 10% . Some forms of OLP, including erythematous and atrophic, are considered more at risk (30), We still do not know if specific clinical/histological features may be predictive of lesions’ transformation into cancer. As a further development of the present research, it would be interesting to evaluate if scoring systems may somehow be correlated to a certain grade of dysplasia and consequently a lower/higher risk of cancer development, so to organize an ad hoc follow up.

The sites, which apparently improved more, were cheeks, tongue dorsum and floor of the mouth for reticulation and cheeks, mandibular gingiva and hard palate for erythema. In general, ventrum of tongue and floor of the mouth had the lowest values. This can be motivated considering that the application of topical steroids in these areas may be penalized by the presence of sublingual and submandibular salivary ducts, which may reduce the persistence of the drug. As regard as statistic significance, erythema resulted as the most frequently improved feature. This is in accordance with the reduction of pain, which is a direct consequence of steroids’ application, which triggers a reduction of inflammation.

On the other hand, evaluations made with WEA-MOD demonstrated a general improvement of patients after steroids but results regarding lesions’ distribution and amelioration of each site after therapy are less clear.

Although WEA-MOD may help estimating the severity of lesions in specific areas of the oral cavity more precisely than the original score, the present study demonstrates it is not very specific. On the contrary, a strength of REU score is that it not only proved effective in estimating OLP severity, but also let you discriminate features of the disease in various areas of the oral cavity, helping the clinician to monitor disease’s and therapy’s activity, but also to monitor risky sites in risky patients with a non- invasive and costless instrument.

Another point of strength demonstrated in the present study is the demonstration that REU score correlates with pain, which can be convenient in terms of therapy and follow up schedule, it allows stratification among patients, thanks to a wide range of scores; it helps identifying predominant characteristics and features of OLP, giving a clear idea of the aspect and evolution of lesions in each patient.

The present study also shows some limits; first, the number of patients, as well as that of the raters should be increased. Second, an ambitious goal could be the association of patients’ score and follow up schedule, which is still not predictable from our results. Eventually, the present study only considers two of the most commonly used scoring systems. A multiple comparison would be more comprehensive and educational.
